# Label-Free Optical Spectroscopy for Early Detection of Oral Cancer

**DOI:** 10.3390/diagnostics12122896

**Published:** 2022-11-22

**Authors:** Siddra Maryam, Marcelo Saito Nogueira, Rekha Gautam, Shree Krishnamoorthy, Sanathana Konugolu Venkata Sekar, Kiang Wei Kho, Huihui Lu, Richeal Ni Riordain, Linda Feeley, Patrick Sheahan, Ray Burke, Stefan Andersson-Engels

**Affiliations:** 1 Tyndall National Institute, University College Cork, T12 R229 Cork, Ireland; 2 ENTO Research Institute, University College Cork, T12 R229 Cork, Ireland; 3 Cork University Dental School and Hospital, Wilton, T12 E8YV Cork, Ireland; 4 Cork University Hospital, T12 DC4A Cork, Ireland; 5 South Infirmary Victoria University Hospital, T12 X23H Cork, Ireland

**Keywords:** oral cancer, Raman spectroscopy, diffuse reflectance spectroscopy, fluorescence spectroscopy, biomarkers, saliva analysis

## Abstract

Oral cancer is the 16th most common cancer worldwide. It commonly arises from painless white or red plaques within the oral cavity. Clinical outcome is highly related to the stage when diagnosed. However, early diagnosis is complex owing to the impracticality of biopsying every potentially premalignant intraoral lesion. Therefore, there is a need to develop a non-invasive cost-effective diagnostic technique to differentiate non-malignant and early-stage malignant lesions. Optical spectroscopy may provide an appropriate solution to facilitate early detection of these lesions. It has many advantages over traditional approaches including cost, speed, objectivity, sensitivity, painlessness, and ease-of use in clinical setting for real-time diagnosis. This review consists of a comprehensive overview of optical spectroscopy for oral cancer diagnosis, epidemiology, and recent improvements in this field for diagnostic purposes. It summarizes major developments in label-free optical spectroscopy, including Raman, fluorescence, and diffuse reflectance spectroscopy during recent years. Among the wide range of optical techniques available, we chose these three for this review because they have the ability to provide biochemical information and show great potential for real-time deep-tissue point-based *in vivo* analysis. This review also highlights the importance of saliva-based potential biomarkers for non-invasive early-stage diagnosis. It concludes with the discussion on the scope of development and future demands from a clinical point of view.

## 1. Introduction

Cancer is a global health problem. According to an estimate of the International Agency for Research on Cancer, World Health Organization, more than 18 million new cancer cases and more than 9.6 million cancer deaths were reported worldwide in 2018 [1], with 19.3 million new cases and 10 million deaths in 2020 alone [2]. Oral cancer is reported to be the 16th most common cancer in the world with a five-year survival rate of only 40–60%, depending on the region of the world [2,3].

Over 90% of oral cancers are squamous cell carcinomas (SCCs), which arise from surface epithelial cells of the oral cavity [4]. Overall survival rates are strongly related to tumour stage at diagnosis [5]. The rates range from 80% for early stage cancers (I/II) to 30–50% in advanced stage cancers (III or IV) [6].

Early diagnosis of oral cancer is critical, both for optimizing survival and for quality of life after treatment. The aim of this review is to assess the current state and progress in oral cancer diagnostic techniques and discuss the importance of optical techniques in oral cancer diagnosis. This review summarizes the importance of body fluids in early detection of oral cancer and the biomarkers that have been investigated in previous studies. In addition, major developments in label-free optical spectroscopy, including Raman spectroscopy (RS), fluorescence spectroscopy (FS), and diffuse reflectance spectroscopy (DRS), are also reviewed. This review concludes with a discussion on the scope of development and future demands from a clinical point of view.

## 2. Diagnosis of Oral Cancer

In the very early stages of oral cancer, the patient may present with a painless flat or discolored lesion on the oral mucosa. Patients, however, more frequently present at a later stage with a mass or non-healing ulcer intraorally, which may be painful and interfere with oral function. Enlargement of the cervical lymph nodes may indicate regional metastasis [7]. Currently, the gold standard procedure for oral cancer diagnosis is the histopathological examination of a biopsy specimen from the tumor site [8]. Biopsy can be an invasive and painful procedure and may require general anesthesia.

Early diagnosis of oral cancer is of clear benefit as the surgical excision of early stage lesions is associated with improved cancer control and survival outcomes, as well as much less morbidity and long-term sequelae for patients. However, one of the challenges with early diagnosis of oral cancer is that many lesions may present as a flat white (leukoplakic) or red (erythroplakic), or exophytic (verrucous) lesions [9]. These lesions may show variable degrees of dysplasia, which is associated with premalignant potential. Diagnosis of early invasive cancer within these lesions by pathological examination relies on the demonstration of invasion through the basement membrane; however, in early stage cancers, this invasion may be focal, thus biopsy of the lesion may miss the cancer if the biopsy is not taken from the part of the lesion corresponding to where the invasion is occurring. Furthermore, such oral premalignant lesions may be extensive, multifocal, or recurrent after initial removal [10]. Therefore, there is a pressing need for a reliable, painless, non-invasive, and cost-effective means of assessment of oral mucosal lesions. Such a technique may facilitate the identification of the most suspicious areas to target for biopsy, selection of oral premalignant lesions that should undergo complete excision, as well as define the extent of surgery for patients with confirmed oral cancers [11]. Table 1 lists a number of detection techniques that are already available and currently being used by clinicians and some that are still in the research phase.

Optical spectroscopy, including Raman spectroscopy (RS), diffuse reflectance spectroscopy (DRS), autofluorescence spectroscopy, and optical coherence tomography (OCT), is able to provide non-invasive point-of-care analysis of structural and biochemical changes during cancer progression [22]. These morphological and biochemical changes in the epithelium serve as important biomarkers in oral cancer detection [23]. The above techniques have a potential to diagnose early stage cancer and dysplasia in real time with high sensitivity, without causing patient discomfort [22,24,25]. These techniques are also effective in analyzing bodily fluids, opening a doorway to oral cancer screening [26,27]. Post screening, non-invasive acquisition of data from multiple locations of an extensive, multifocal, and heterogeneous premalignant lesion may assist in performing an informed biopsy.

## 3. Optical Spectroscopy for Oral Cancer Screening *Ex Vivo*

When light interacts with a biological matter, a combination of different optical phenomena such as absorption, reflection, scattering, and fluorescence takes place. Provided that optical properties are known, spectroscopic analysis of these optical processes can provide information about the physical, chemical, and metabolic state of the tissue. Spectroscopy can be classified either by the energy of electromagnetic wave used or based on their interaction with the biological samples. Among the wide range of spectroscopic techniques, Raman spectroscopy, fluorescence spectroscopy, and diffuse reflectance spectroscopy have the ability to provide biochemical information in real-time, point-based diagnosis [28,29,30]. Raman, an inelastic scattering technique, probes the molecular vibration, providing information on conformational and compositional changes in the sample under investigation. In conventional Raman set-up, the monochromatic light interacts with the superficial layer of the sample. In contrast, DRS is implemented with a spatial separation between the illumination and detection fiber to enhance the collection of optical properties (absorption and scattering) of the sample in depth. For DRS, a specific optical window (650–950 nm) is preferred to avoid the excessive attenuation of light owing to dominating chromophores such as haemoglobin and water. Table 2 below represents some subjective views of the general characteristics of Raman spectroscopy, diffuse reflectance spectroscopy, and fluorescence spectroscopy for diagnostic support of oral cancers.

Timely therapeutic intervention also requires technological developments that enable the detection of biochemical changes in bodily fluids and assist in biopsy. In this regard, biofluid spectroscopy is a potential method for non-invasive cancer screening and diagnostics [35]. Raman spectroscopy has the upper hand in the study of biofluids in situ as water is a very weak Raman scatterer, as described in Table 2. There are many studies available on detecting cancer-associated biochemical changes using Raman spectroscopy in saliva [36,37], urine [38], cervical fluid [39], and blood plasma [40] with this technique [41].

The idea of conducting a simple saliva test in order to diagnose oral malignancies is extremely appealing. It offers convenient, inexpensive, and non-invasive sample collection as compared with blood and tissue biopsies. Saliva is a fluid with a very complex composition. Its major component is water—about 98%—with enzymes, electrolytes, minerals, proteins, nucleic acid, antibodies, or nasal secretions dissolved in it. It may also contain bacteria, viruses, epithelial cells, blood, or its derivatives from lesions and other biomarkers [42,43]. Its composition can represent the condition of the whole body and, therefore, it can be a very effective diagnostic medium for many diseases, particularly those in the oral cavity. In addition, molecular biomarkers present in saliva have the potential for patient stratification. In a recent study, the potential of fluorescence spectroscopy has been discussed to differentiate between OSCC, dysplasia, and control group using saliva as a specimen. This study reports significantly different fluorescence intensities associated with flavin adenine dinucleotide (FAD) and porphyrin among three groups [44]. With advancements in nanotechnology and medical science, many biomarkers associated with oral cancer have been reported, but still it is a challenge to confirm “true” biomarkers that can be identified non-invasively with high sensitivity and specificity. Previous studies show that saliva is a more effective diagnostic medium in the early stages of oral cancer than plasma owing to direct contact to the pathology site [43]. In contrast, serum could serve as a good diagnostic specimen for late stage oral cancer [45]. Another factor to consider is the reduced production of saliva in patients after treatment (surgery or radiotherapy), rendering it difficult to collect salivary specimens in follow-up patients. In a more recent study, saliva samples collected from 148 participants were subjected to Raman spectral analysis for patient stratification in healthy volunteers, tobacco habitués, and oral cancer patients. This study confirmed the identification of spectral differences among three groups [46]. In another study, Koster reported diagnostic performance accuracy of 91.7% in a cohort of head and neck cancer and benign control by unifying Raman spectra of plasma and saliva sample of each participant to assess metabolite composition [47]. Some important salivary biomarkers for oral cancer are shown in Table 3.

## 4. Optical Spectroscopy for *In Vivo* Diagnosis

Cancer progression is a complex process in which molecular and cellular changes accumulate over time and lead to transitions of tissue from normal to malignant. The morphological and biochemical changes in the cancerous tissue such as epithelium thickening, enlargement of nucleus, and changes in the extracellular matrix (ECM) architecture act as important biomarkers in oral cancer detection [23]. Over the past few decades, optical spectroscopic techniques have shown great potential in tissue differentiation including cancer identification via probing these chemical changes. Further, the advent of new optical and data analysis techniques has provided the platform to integrate multiple spectroscopic modalities, which enables the collection of complementary information to improve the accuracy of the cancer differentiation.

Raman spectroscopy can inform the chemical signature of the tissue and provides structural and chemical composition of the targeted analytes. It relies upon inelastic scattering of light typically within the visible and NIR region. Changes in the concentration of biomolecules or any mutation in the tissue during cancer progression provide notable changes in Raman signals [81,82,83]. A prominent discriminating feature among healthy tissue and different stages of malignancy is a change in the concentration of proteins, lipids, and nucleic acid in the epithelium and decreased collagen with inflammation in connective tissue [84]. Recently, a software tool has been reported for the bimolecular composition analysis through Raman spectra [85,86]. Previous Raman spectra-based studies differentiated *in vivo* tissues and in vitro tissue samples using different classification models such as principal component analysis (PCA) [87], support vector machine (SVM) [88], quadratic discriminate analysis (QDA), and linear discriminant analysis (LDA) [28].

Fluorescence spectroscopy can detect metabolic changes in the tissue associated with enhanced metabolic activity and redox imbalance [89]. Such alterations cause an increase in the production of nicotinamide adenine dinucleotide phosphate (NADPH) and other metabolites [90]. This spectroscopy involves using a UV/visible light beam to excite the molecules of some particular tissue compounds—fluorophores. Following the excitation, the molecule quickly returns to a low vibrational energy level through a non-radiative vibrational relaxation. From this level, the molecule can then return to its ground state by emitting the excess energy in the form of a visible photon-fluorescence. When illuminated with an appropriate wavelength, biological tissue fluoresces because of the presence of many endogenous fluorophores including NADPH, NADH, collagen, flavins, elastin, and porphyrins [90,91]. The concentration of these fluorophores changes as the cancer progresses and can thus act as a biomarker for malignancy. Some of the fluorophores have overlapping excitation and emission spectra; however, they are more or less distinguishable by appropriate selection of excitation and emission wavelengths, which facilitates the quantification of various tissue fluorophores. The spectroscopic method is thus necessary to extract concentrations of particular fluorophores and provide a diagnostic value. If the tissue autofluorescence does not provide sufficient diagnostic accuracy, one can employ a contrast agent in the form of an exogenous fluorophore.

Diffuse reflectance spectroscopy (DRS) is based on the light that is backscattered multiple times inside the specimen and contains information about its optical properties. It can be used to extract both concentrations of absorbing tissue chromophores and morphological changes occurring in the sample. Similar to the fluorophores, chromophores can be utilized to indicate malignancy. Morphological alterations such as the uncontrolled growth of neoplastic cells or enlarged nuclei will influence the scattering properties and DRS can be employed to extract such diagnostic information efficiently. Scattering in tissue is quite complex and is linked to local changes in the refractive index. The overall change in the refractive index, shape, size, density of cellular structures, and absorption determines the spectral features of back-scattered back reflected light. DRS can operate in the UV, visible, and NIR regions. By controlling the probe configuration, one can obtain the signal from the superficial epithelial layer or from the deeper tissue layers. In a recent study, Wang et al. demonstrated the potential of a hand held DRS probe through in-depth characterization for *in vivo* diagnosis of oral diseases in a real-time clinical environment [92].

**Figure 1:**
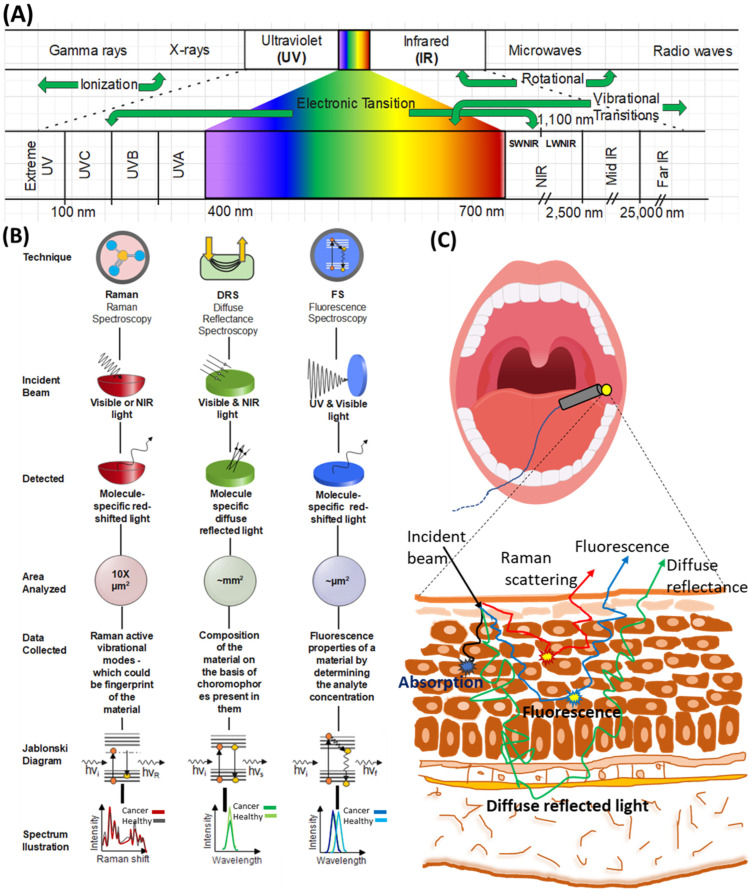
(**A**) Electromagnetic spectrum and light tissue interaction. (**B**) Key features of Raman spectroscopy (RS), diffuse reflectance spectroscopy (DRS), and fluorescence spectroscopy (FS). (**C**) Light tissue interaction and comparative depth of RS, DRS, and FS in oral mucosa.

Furthermore, *in vivo* optical spectroscopy for disease diagnosis would allow clinicians to identify cancerous lesions based on spectral markers and significantly reduce the burden of biopsies. In this case, incident light is delivered to the tissue through a fiber optics probe placed in an endoscope in a localized manner. There has been significant development in this field in the last few decades. Figure 1, created with Edraw Max software, represents some typical features of the above-mentioned spectroscopic techniques.

## 5. Multimodal Spectroscopy Approach

Multimodal spectroscopy (MMS) refers to the production of signal simultaneously from multiple spectroscopy techniques. The goal of this approach is to further improve the early detection and localization of malignancies by measuring several biomarkers. In this case, the progression of various cellular events during cancer can be examined all together and in real time. Such an approach could be a promising tool to provide complementary, depth-sensitive information.

A multimodal instrument was designed by Greening et al. to monitor the progression of dysplasia in the oral cavity. This system incorporated high-resolution fluorescence imaging and sub-DRS (sub-diffuse reflectance spectroscopy). This system used two light sources—a halogen lamp for the sub-DRS modality and a 455 nm LED for fluorescence imaging. Two sets of liquid phantoms were designed to produce and validate the look-up table (LUT)-based inverse model. The sampling depth was also calculated for both probes. Subsequently, the system was validated in the clinical environment and *in vivo* sub-DRS data were collected from the inner lip of 13 volunteers. This hybrid system was reported to be capable of gathering information on functional and structural properties of tissue [93].

In a detailed real-time *in vivo* study by Lin et al., autofluorescence, DRS, and Raman spectroscopy were used in combination with white light imaging guided endoscopy for the detection of nasopharyngeal cancer. Sixty volunteers were involved in this study, 30 of whom were suffering from nasopharyngeal cancer confirmed by a biopsy immediately after the endoscopy. It was possible to distinguish between normal and cancer tissue with a very high sensitivity of approximately 98% and specificity of approximately 95% using a PCA-LDA diagnostic algorithm. It was reported that, as compared with white light imaging (WLI), autofluorescence imaging was much better at providing information on the metabolic state and biochemical composition of tissues. When normal tissue develops a malignancy, morphological changes take place in tissue such as thickening of epithelium, the extracellular matrix is decreased, cellular metabolic fluorophores such as flavins and nicotinamide adenine dinucleotide are reduced, and the concentration of hemoglobin is increased. As a consequence of the absorption of blue light by haemoglobin, a reduction in the amount of green-emitting metabolic fluorophores, and re-absorption of fluorescence by the thickened epithelium, malignant tissues tend to exhibit an overall decrease in the green autofluorescence. So, when normal tissue in the oral cavity is illuminated by a blue light, healthy tissue appears green, but as the cancer progresses, there is a gradual decrease in green fluorescence, but a proportionally less decrease in red fluorescence, hence cancerous tissue appears dark [94]. Lin et al. reported that autofluorescence imaging (AFI) helps in localizing the lesion more accurately than WLI. It also sets a ground for Raman spectroscopy and DRS to provide better guidance. They developed an integrated endoscopy system with four modalities including WLI, AFI, DRS, and Raman spectroscopy to achieve a high specificity by studying the biomolecular changes in malignant tissues. The Raman spectroscopy results showed a lower concentration of phospholipids in malignant tissue as compared with the normal surrounding tissue. However, an increased concentration of protein in α-helix, phenylalanine, and nucleic acid was observed in cancer tissue. The reflectance data were observed in the range 400–700 nm. Throughout this spectral range, the intensity of the reflected signal was higher for the normal tissue as compared with the malignant tumors, with this change becoming more prominent after a 600 nm wavelength. Detected valleys at 540 and 580 nm also represent less oxygenated hemoglobin in cancer tissues [81]. In another study by Jermyn et al., which combines DRS, Raman, and intrinsic fluorescence spectroscopy for detection of colon, lung, brain, and skin cancers, sensitivity and specificity were reported to be >99% and 93%, respectively. It was also reported that the detection capability of that system was not dependent on the cancer type, but that the system could classify the tissues on the basis of spectral features for all malignancies studied [26].

*In vivo* optical spectroscopy can also facilitate clinicians to differentiate among benign lesions and severe dysplasia and provide guidance for tumour classification. In one study on Raman spectroscopy performed on paraffin preserved tissue samples, Ibrahim et al. reported accuracy rates of 80–90% in the distinction of mild, moderate, and severe dysplasia in different tissue types of the same person and 60% accuracy in an interpatient study based on spectral markers. They also reported 70–80% accuracy in differentiating SCC from dysplasia and benign lesion in different tissues [95]. Furthermore, Jaychandran et al. managed to distinguish oral malignancy and premalignancy from normal control group with an accuracy of 97.4% for tissue and 93.1%, 90.5%, and 78% for saliva, urine, and blood samples, respectively [96]. In a more recent study, Li et. al. tried to discriminate OSCC, dysplasia, and healthy mucosa by analyzing biochemical variations at different stages with near infrared Raman spectroscopy in order to evaluate the performance of different classification models such as PCA-LDA and SVM. They reported relatively higher classification accuracy for the SVM model as compared with PCA-LDA. It was also observed that oral dysplasia and OSCC have a higher content of DNA and protein than normal mucosa. However, there was no significant change in the Raman signal of various dysplastic grades [88].

In an in vitro study, Nour et al. used gold nanoparticles for detecting SCCs of the tongue through diffuse reflectance analysis and laser-induced fluorescence with 91% accuracy [97].

Tobacco usage increases the risk of damage to mucosa and the genetic changes ultimately cause abnormal cell growth. Identifying these changes may help to diagnose the lesion in the early stages. Shaiju et al. reported the importance of fluorescence spectroscopy in combination with multivariate analysis to analyze pathological changes due to tobacco abuse in the early stages. They reported that total porphyrin and hemoglobin levels in smokers are comparable to those with leukoplakia [98]. In a clinical study on thirty patients with erythroplakia and leukoplakia, it was reported that induced fluorescence in tumor could improve diagnostic accuracy. The aim of the study was to distinguish between normal and potentially malignant oral mucosa by fluorescence spectroscopy. The study reports lower fluorescence signal from the tumor center as compared with tumor borders. Similar behaviour was observed in erthroplakic lesions that have low fluorescence in comparison with normal tissue. A higher hemoglobin content could be the reason for the reduced fluorescent signal in both of these scenarios, as hemoglobin is an endogenous absorber of light. This study also points out differences in spectral features of normal patient mucosa and healthy volunteer mucosa [99,100]. Overall, the published literature highlights the potential of the spectroscopic techniques in differentiating benign premalignant lesions from invasive malignant lesions with adequate accuracy, which can be improved further by integrating multiple modalities into a system.

The importance of multimodal instruments can never be denied in medical diagnostics and there is still room for further developments. There are other published studies involving multimodal optical techniques to detect atherosclerotic plaque [101], amyloid plaques [102], breast cancer [103], and non-melanoma skin cancer in real time [104]. Now, it is time to incorporate other techniques and imaging modalities with spectroscopy for the diagnosis of oral cancer in the early stages and in real time.

## 6. Discussion

Ranking as the 16th most frequently diagnosed cancer in the world [2,3], oral cancer still has a very poor prognosis [6,105] and a high rate of recurrence [106,107]. Cancer stage affects the treatment outcome. In the past few decades, many efforts have been made to develop optical spectroscopy as an assisting tool for targeted and informed biopsy as well as a guiding tool to identify surgical margins more precisely. This article provides an overview of the potential of optical spectroscopic techniques in detecting early stage oral cancer. All of the spectroscopic techniques included are green in the sense they do not employ any exogenous substances or contrast agents [108]. Raman, diffuse reflectance, and fluorescence spectroscopy are new emerging spectroscopic techniques and hold promise to facilitate timely treatment to cure a higher fraction of the oral cancers by detecting them in the earlier stages. DRS achieves better results in tissue classification roughly between 400 nm and 700 nm. Fluorescence spectroscopy allows signal detection from a very low amount of fluorophore and can achieve a sensitivity of more than 80 % in staging cancers. Because of limited chemical specificity, it is, however, sometimes hard to discriminate overlapping signals from different fluorophores, compromising the diagnostic accuracy. Moreover, not all of the biological tissue structures are fluorescent when excited using visible or NIR wavelength. On the other hand, all biomolecules have their own specific Raman signatures, which can be probed by a single excitation wavelength. It complements mid-IR spectroscopy by detecting vibrational modes that are mid-IR inactive. It is not heavily affected by water molecules and is suitable for aqueous solutions and biological specimens. In fact, in a study on 14 patients undergoing tongue resection, Barosso et al. discriminated between healthy and cancerous tissue in the oral cavity on the basis of water content with an accuracy of 99% and specificity of 92% [109]. Raman spectroscopy along with NIRS and mid-IR spectroscopy comprise the “three sisters” of vibrational spectroscopy [108]. In NIR spectroscopy, excitation to higher vibrational states within the ground electronic state of a molecule leads to overtone and combination vibrations [110]. While Raman spectroscopy performs very well for symmetric bonds, NIRS only works for asymmetric bonds. Fluorescent spectroscopy, DRS, and Raman spectroscopy are valuable and important technologies with complementary pros and cons. Together, they can give rise to a powerful tool for effective diagnosis. Robust machine-learning-based models can be explored to fuse the informative features from the multimodal system, improving the classification and/or prediction accuracies.

## 7. Limitations and Future Outlook

Despite all of the advances and research, optical spectroscopies are still far from being conventionally used in clinical diagnostics for oral cancer because of instrumental limitations and lack of diagnostic validation. Each spectroscopy has its own drawbacks. Raman spectroscopy experiences strong background fluorescence and inherently weak signal. Excitation light could be in the UV, visible, and NIR regions and hence there is the need for system design, optimization, and validation for a change in Raman excitation wavelength. The UV and visible range is used for resonance enhanced Raman spectroscopy of proteins and porphyrin like molecules where enhanced signals dominate over the fluorescence background. However, for *in vivo* Raman, the NIR range is preferred to avoid excessive fluorescence background and to improve the depth of penetration. In SERS, although the signal is strong, there remain clarifications regarding how to best incorporate the metallic nanoparticles in terms of size and overall geometry to secure reproducible signals and limit toxicity. DRS signals are not linearly dependent on chromophore concentrations, making direct correlation between spectral features and diagnostic interpretation more complex, and the technique typically requires big datasets to ensure diagnostic accuracy. The biggest drawback of fluorescence spectroscopy is the risk of misdiagnosis owing to the reported somewhat lower specificity. Therefore, there is still the need for more research to incorporate different optical spectroscopies into clinical practice.

During the last few decades, optical spectroscopy has experienced substantial advancements, which can be categorized as instrumentation development and analysis approach maturation. The majority of these developments have pushed the boundary of possibilities, taking this family of techniques to a new level and redefining it. However, considering the current status of advancement, there is still the need for improvement in instrumentation, analysis approach, and standardization.

### 7.1. Instrumentation

Recently, instrument development is being driven in four directions; that is, miniaturization and clinical adoption, instrument sensitivity, multimodality, and further technique sophistication. There is still room for development in all of these directions, especially considering the final user will be a clinician. Miniaturized, hand-held, and portable spectrometers enable users to bring the laboratory to the sample in point-of-care diagnostics. A simplified and concise workflow to familiarize the clinician and combination of portable handheld system with sophisticated machine learning models that can provide real-time output in a language understandable to clinicians is required. Adequate training protocols need to be established learning from on-going spectroscopic clinical trials across the world. Various optical societies are working on standardizing these protocols and making them easier for the clinicians to understand. Apart from miniaturization, disposable probes, cost efficiency, and an easy approach are also necessary. In the current scenario, each modality requires different light sources and spectrometer/detectors, which makes it expensive. However, system miniaturization and integration can still make the technology attractive from both a design/footprint and a cost perspective. Additionally, the new developments in the instrumentation area provide a hope to make it more cost effective. Increased instrument sensitivity and capability can enable new applications. The combination of different techniques may also open new dimensions in the medical science. Apart from combining multiple complementary modalities, one can also develop techniques that are more sophisticated and provide higher diagnostic accuracy. For example, standard imaging techniques lack sufficient spatial resolution and sensitivity. A possible solution to these limitations could lie in non-linear microscopy or hyperspectral imaging. Another possibility could be to employ time-domain Raman spectroscopy. Multiscale and multimodal imaging techniques that combine biochemical macroscopic imaging to morphological subcellular imaging techniques could also lead to new pathways in clinical research. Incorporating fluorescence with Raman into a single process might provide single molecule sensitivity with the specificity of Raman spectroscopy and open a new era in medical diagnostics. Tagging or incorporation of nanoparticles with these imaging modalities can also play an important role in improving the sensitivity of these techniques and the signal-to-noise ratio. In addition to this, the use of multiple wavelengths for excitation can provide more hidden information. Thus, future studies should focus on combination of optical spectroscopic techniques and multiple wavelengths to overcome the shortcomings of a single technique. Another possible future improvement could be automation of these techniques, which can make possible the analysis of multiple specimens simultaneously.

### 7.2. Analysis Approach/Data Analysis

Experiments have shown that spectral techniques are a valuable source of large amounts of clinical data. Years of research and technical development have provided a large number of overlapped datasets that are difficult to interpret. These data could provide useful information in medical diagnostics and potentially lead to a future paradigm shift. Various groups are making efforts to translate these spectral data into clinically relevant information. Currently, there are three general ways to translate the available spectral data. The first approach that does not require any prior data processing and knowledge of light tissue interaction is the direct analysis of raw spectral data using supervised and unsupervised machine learning methods, such as neural network, component analysis, and clustering [111]. A second method, which requires expert knowledge, involves the translation of spectral data into optical parameters such as absorption or scattering coefficient at different wavelengths and then correlating those with classically diagnosed tissue type based on statistical analyses that are more conventional [112]. A third method, which is even more complicated, includes the transformation of these physical properties into relevant biological parameters associated with molecular vibrations, chromophores, and fluorophores in tissue or other biomarkers. This approach requires detailed knowledge of biological substances within the tissue that absorb or scatter light [112,113]. Any of these three approaches can be adopted and are acceptable for clinical use if it helps the clinician to interpret the biological information hidden in the data and recognize a tissue abnormality at an early stage. The analysis can be furthered by incorporating new techniques such as explainable machine learning and artificial intelligence algorithms for better data classification efficiency and a reduced error rate. These more novel techniques can help to design an intelligent user-friendly real-time monitoring system or automatic recognition of malignancy and tumour subtypes.

### 7.3. Requirement of Standardization

Optical spectroscopy is still a technique under development and reliable standardization of the techniques is required along with advancement in hardware technology and analysis tools. Currently, there are different diagnostic methods, instruments (spectroscopes, probes, and so on) and analytical techniques, which makes it difficult to compare the results from different studies and, thereby, to fully assess the diagnostic capabilities and importance of the technology. To obtain accurate results, a standardized calibration procedure is necessary. There are few attempts to standardize the diffuse optical methods using phantoms. However, similar standardization methods need to be developed and adopted for label-free optical spectroscopy. In addition, awareness of the use of standards is required to accelerate the development of novel techniques and widespread adoption in regular clinical practice [113,114].

## 8. Summary and Conclusions

Biomedical optical spectroscopy provides a deep intuitive understanding about the light–tissue interaction and provides opportunities for real-time diagnostic information. The modalities including Raman, diffuse reflectance, and fluorescence spectroscopy are able to provide chemical and structural properties of analyzed biological specimens non-destructively. These optical spectroscopic techniques can facilitate the identification of lesions, which do not require biopsy owing to low or no risk of malignant progression and can help in targeted biopsy of the biologically most advanced area from within leukoplakic, erythroplakic, or verrucous lesions, especially lesions that are extensive and/or multifocal. Optical spectroscopy offers a potential mean of non-invasive diagnostics. In situ optical spectroscopic detection through endoscope is also capable of identifying malignant and premalignant lesions and has the potential to classify different tissues in real time. It can enhance a surgeon’s vision by providing efficient diagnostic information and maximizing the chances of successful tumor removal with minimal harm to the surrounding healthy tissue. Body fluids, especially saliva, can serve as a very useful specimen for the early detection of oral cancer. Optical spectroscopy of blood and its components also has proven potential in the diagnosis of gastrointestinal and oral cancer. Multimodal approaches and incorporating optical modalities with other biological techniques can yield clear benefits by overcoming the limitations of individual techniques. Different mathematical models to analyze multispectral data could also serve an important role in this regard. Finally, standardization is necessary for translating these optical spectroscopic approaches and modalities into clinical diagnostic practice.

## Figures and Tables

**Table 1:** Techniques for oral cancer diagnosis in addition to clinical oral examination.

Diagnostic Techniques		*In Vivo*	Non-invasive	Real Time	Time Required *	Label-Free	Non-ionizing	Portability	Spatial Resolution	Detection Accuracy	Cost-Effectiveness **	References
Histopathology	Excisional biopsy	✕	✕	✕	days	✕	✓	✕	µm	High	Low cost	[12,13]
	Incisional biopsy	✕	✕	✕	days	✕	✓	✕	µm	High	Low cost	
	Brush biopsy	✕	✓	✕	days	✕	✓	✕	µm	High	Low cost	
	Punch biopsy	✕	✕	✕	days	✕	✓	✕	µm	High	Low cost	
Vital staining	Toluidine blue	✕	✕	✕	days	✕	✓	✕	µm	High	Low cost	[14]
	Iodine staining	✕	✕	✕	days	✕	✓	✕	µm	High	Low cost	
	Methylene blue	✕	✕	✕	days	✕	✓	✕	µm	High	Low cost	
	Lugol’s iodine	✕	✕	✕	days	✕	✓	✕	µm	High	Low cost	
	Acetowhite staining	✕	✕	✕	days	✕	✓	✕	µm	High	Low cost	
	Double staining	✕	✕	✕	days	✕	✓	✕	µm	High	Low cost	
Imaging techniques	OCT (Optical coherence tomography)	✓	✓	✓	10–20 min	✓	✓	✕	µm	Medium	Expensive	[15,16,17]
	CT scan (computed tomography)	✓	✓	✓	10–20 min	✕	✕	✕	Mm	Medium	Expensive	
	PET scan (positron emission tomography)	✓	✓	✓	45–60 min	✕	✕	✕	Mm	Medium	Expensive	
	MRI (magnetic resonance imaging)	✓	✓	✕	15–90 min	✓	✓	✕	Mm	Low	Expensive	
Molecular analysis	Immunohistochemistry	✕	✕	✕	days	✕	✓	✕	µm	Very high	Low cost	[18,19,20]
	In situ hybridization	✕	✕	✕	days	✕	✓	✕	µm	Very high	Low cost	
	Flow cytometry	✕	✕	✕	days	✕	✓	✕	µm	Very high	Low cost	
	Mass spectrometry	✕	✕	✓	s	✓	✕	✕	NA	Very high	Low cost	
	PCR (polymerase chain reaction)	✕	✓	✓	min	✓	✓	✓	NA	Very high	Low cost	
Light-based detection	Tissue fluorescence (autofluorescence imaging, Velscope, Identafi)	✓	✓	✓	s	✓	✓	✓	Low	Low	Low cost	[12,15,21]
	Chemiluminiscence (ViziLite, Microlux/DL, MCE)	✓	✓	✓	s	✕	✓	✓	Low	Low	Low cost	
	Optical spectroscopy	✓	✓	✓	s	✓	✓	✓	Low	Medium	Low cost	

* Time required to obtain the results after the sample collection. ** Cost/visit. NA, not available.

**Table 2:** General characteristics of RS, DRS, and FS in cancer diagnosis.

	Raman Spectroscopy	Diffuse Reflectance Spectroscopy	Fluorescence Spectroscopy
Basic Phenomenon	Scattering	Scattering and absorption	Photoluminescence event
Incident light	Ultraviolet, visible, NIR	Visible, NIR	Ultraviolet, visible
Detected light	Inelastically scattered Raman-shifted light	Diffusely scattered light	Emission from endogenous fluorophores
Spectral range	10–4000 cm^−1^	200–3000 nm	360–700 nm
Typical tissue depth probed	<0.1 mm [31,32]	<1.5 mm [33]	0.1–10 mm [34]
Label-free/noninvasive/nondestructive	Yes	Yes	Yes
Real time data acquisition	Yes	Yes	Yes
Suitable for *ex vivo*/*in vivo* analysis	Yes	Yes	Yes
Photo-bleaching	No	No	Yes
Suitable for biological specimens	Yes	Yes	Yes
Suitable to incorporate with endoscopy	Yes	Yes	Yes
Suitable for water-based specimens, blood, and saliva	Yes	Only highly scattering specimens (selective wavelength regions)	Yes
Special sample preparation	No	No	No
Specificity (bandwidth)	High	Moderate	Moderate
Sensitivity	Low	High	High
Nanoparticles	Not required but can be used —SERS	Not required	Not required—can be used in SPR-enhanced fluorescence
Possible biomarkers	Lipids, protein, nucleic acid, circulating tumor cells	Water, lipid, collagen, deoxy and oxyhaemoglobin	Endogenous fluorophores—such as NADH, FAD, collagen, and porphyrins

**Table 3:** Important salivary biomarkers for oral cancer.

Category	Biomarker	Healthy	Oral Cancer	Significance	Trend	Ref.
Proteins and amino acids	Phenylalanine	0.011 µmol/mL	0.105 µmol/mL	*	Increase	[48]
		4100 ng/mL	T1, T2 = 2500 ng/mL; T3, T4 = 1900 ng/mL	*	Decrease	[49]
	Tyrosine	0.112 µmol/mL	0.343 µmol/mL	*	Increase	[48]
	Tryptophan		3.81 ± 0.62 µM			[50]
	Leucin	0.015 µmol/mL	0.241 µmol/mL	*	Increase	[48]
		2300 ng/mL	T1, T2 = 600 ng/mL; T3, T4 = 500 ng/mL	*	Decrease	[49]
	Alanine	0.096 µmol/mL	0.178 µmol/mL	*	Increase	[48]
	Valine	0.038 µmol/mL	0.165 µmol/mL	NS	Increase	[48]
	Isoleucin	0.033 µmol/mL	0.236 µmol/mL	*	Increase	[48]
	Aspartic acid	0.035 µmol/mL	0.241 µmol/mL	*	Increase	[48]
	Serine	0.050 µmol/mL	0.187 µmol/mL	*	Increase	[48]
	Glycine	0.065 µmol/mL	0.288 µmol/mL	*	Increase	[48]
	Threonine	0.157 µmol/mL	0.435 µmol/mL	NS	Increase	[48]
	Arginine	0.047 µmol/mL	0.220 µmol/mL	*	Increase	[48]
	Isoleucine	0.033 µmol/mL	0.236 µmol/mL	*	Increase	[48]
	Methionine	0.012 µmol/mL	0.162 µmol/mL	*	Increase	[48]
	Albumin	0.17–0.36 g/L; mean 0.24 g/L	0.192–0.67; mean 0.36 g/L	*	Increase	[51]
		0.2 ± 0.1 mg/mL				[52]
		0.28 ± 0.19 g/dL	0.82 ± 0.41 g/dL	*	Increase	[53]
		0.8–192 mg/dL				[52]
	α-amylase	3257 ± 1682 U/mL				[52]
		65.2 mg/mL	68.07 mg/mL		Increase	[54]
		1080 ± 135.6 IU/L				[52]
	Interlukins (IL-8)	250 pg/mL	OSCC 720 pg/mL	*	Increase	[55,56]
	Interlukins (IL-6)	0 pg/mL	OC 86.5 pg/mL	*	Increase	[56]
		16 ± 3.91 ^#^ pg/mL	129 ± 66.59 ^#^ pg/mL	*	Increase	[57]
	Osteopontin	35.1 ng/mL	39.23 ng/mL		Increase	[56]
	CRP (inflamation marker)	0.05–61 µg/L				[58]
	suPAR	5.22–28.1 ng/mL				[58]
	Survivin	2.44 ± 4.22 pg/mL	8.69 ± 10.15 pg/mL	*	Increase	[59]
	Kallikrien 5	~6 pg/mL	~12 pg/mL	*	Increase	[60]
	Cathepsin	9–18 * ng/mL				[61]
	Cathepsin V	~8 pg/mL	~14 pg/mL	*	Increase	[60]
	lactate dehydrogenase (LDH)	3.833 ± 1.1044 U/L	99.83 ± 49.33 U/L	*	Increase	[62]
		63.04 ± 47.4 mg/dL	1515.17 ± 765.14 md/dL		Increase	[63]
	(Endotheline-1) ET-1	0 to 9.629 fmol/m	0 to 7.554 fmol/mL	NS	Increase	[64]
		0.506–19.280 pg/mL; 4.5299 ± 3.7380 pg/mL	2.140–52.229 pg/mL; 13.51 ± 14.15 pg/mL	*	Increase	[65]
	Statherin	0.5–4.0 μg/mL; mean 0.96 µg/mL				[66]
		4.3–5.59 µM; mean 4.93 ± 0.61 µM	0–6.45 µM; mean 2.28 ± 2.86 µM	*	Decrease	[67]
	Carbohydrate antigen (CA 125)	137.12 ± 124.58 U/mL	498.10 ^#^ U/mL; 19.9–1312.32 U/mL	NS		[68]
		33.00 ± 24.37 mg/dL	888.15 ± 306.1 mg/dL	*	Increase	[63]
	Tissue polypeptide-specific antigen (TPS)	96.20 ± 71.60 U/mL	272.28 U/m ^#^; 13.61–4706.17 U/mL	NS		[68]
	CD 44	1.09 ng/mL	7.85 ng/mL	*	Increase	[69]
Antioxidants	Glutathione	9.4 µmol/dL	8.2 µmol/dL		Decrease	[70]
	vitamin c	0.925 mg/dL	0.4787 mg/dL	*	Decrease	[71]
Carbohydrates	Fucose	0.38–17.0 mg/dL mean 2.94 mg/dL	Pre-cancer 0.112–18.46 mg/dL; mean 7.02 mg/dL	*	Increase	[72]
			OSCC 0.11–30.60 mg/dL; mean 11.66 mg/dL			
		3.19 ±1.94 mg/dL	6.14 + 2.16 mg/dL	*	Increase	[73]
		3.18 mg/dL	11.66 mg/dL	*	Increase	[72]
	Sialic acid	0.134–0.311; mean 0.189 mmol/L	0.140–0.336; mean 0.22 mmol/L	*	Increase	[73]
		21.65 ± 5.71 mg/dL	204.85 ± 60.38 mg/dL	*	Increase	[74]
		1.35 ± 1.53 mg/dL	5.30 ±1.45 mg/dL	*	Increase	[73]
Lipids	Linoleic acid	339.3 ± 267.9 ng/mL	1092.3 ± 1927.8 ng/mL		Increase	[75]
	15-HETE (Hydroxyeicosatetraenoic acids)	0.4 ± 0.8 ng/mL	5.4 ± 6.8 ng/mL	*	Increase	[75]
	Arachidonic acid	32.6 ± 26.6 ng/mL	606.9 ± 1695.7 ng/mL		Increase	[75]
	Lipo per oxidation products (MDA)	6.15–9.06 nmol/mL; mean 6.92 nmol/mL	5.56–7.78 nmol/mL; mean 6.58 nmol/mL	NS		[51]
Other biomarkers	Pyruvic acid	1.32 ± 0.10	3.49 ± 0.47	*	Increase	[76]
	Uric Acid	25.2–161.2 nmol/mL; mean 76.8	43.2–182.9; mean 93 nmol/mL	*	Increase	[51]
	Urea	4.35–8.78 mmol/L; mean 6.76 mmol/L	4.62–11.29; mean 8.66 mmol/L	*	Increase	[51]
	Total protein	0.78–1.53; mean 1.11 g/L	0.47–1.59 g/L; mean 0.84 g/L	*	Decrease	[51]
		0.47 ± 0.19 mg/mL				[52]
		0.9 ± 0.2 mg/mL				[52]
		43–710.0 mg/dL				[52]
		1.07 ± 0.59 mg/mL	1.01 ± 0.43 mg/mL	NS		[77]
		2.67 ± 0.54 mg/mL				[52]
	CYFRA-21-1	3.06 ng/mL	17.46 ± 1.46 ng/mL	*	Increase	[78]
	Basic fibroblast growth factor (bFGF)	3.17 ± 0.43 pg/mL	8.80 ± 1.26 pg/mL	*	Increase	[79]
		0.3 ± 0.3 pg/mL	OLP patients 5.9 ± 2.9 pg/mL	NS	increase	[80]

* Significant difference, NS, non-significant difference, ^#^ median.

## Data Availability

Not applicable.
